# CD45RO+TILs: cellular biomarkers for larynx squamous cell carcinoma outcome

**DOI:** 10.1016/j.bjorl.2022.09.007

**Published:** 2022-10-14

**Authors:** Yousef Mohammadi, Simin Ahmadvand, Maryam Mirtalebi, Mohammad Javad Ashraf, Bijan Khademi, Abbas Ghaderi

**Affiliations:** a Shiraz University of Medical Sciences, School of Medicine, Department of Immunology, Shiraz, Iran; b Shiraz University of Medical Sciences, School of Medicine, Shiraz Institute for Cancer Research, Shiraz, Iran; c Shiraz University of Medical Sciences, School of Medicine, Department of Pathology, Shiraz, Iran; d Shiraz University of Medical Sciences, Otolaryngology Research Center, Department of Otorhinolaryngology, Shiraz, Iran

**Keywords:** Head and neck squamous cell carcinoma, Tumor infiltrating lymphocytes, Prognostic marker, CD45RO+ memory cells, Larynx squamous cell carcinoma

## Abstract

**Objective:**

The prognostic importance of Tumor-Infiltrating Lymphocytes (TILs) in the tumor microenvironment of various cancers is increasingly recognized. In the present study, we aimed to investigate the prognostic value of CD3+, CD4+, CD8+, and CD45RO + TILs and their relation to histopathological features in larynx squamous cell carcinoma.

**Methods:**

Formalin-Fixed and Paraffin-Embedded (FFPE) samples from 63 primary larynx squamous cell carcinoma patients were immunostained for CD3, CD4, CD8, and CD45RO expression. Positive cells in micrographs from Invasive Margin (IM) and Tumor Center (CT) of tissue specimens counted by ImageJ software and their correlation with disease outcome were analyzed.

**Results:**

The expression level of TILs subpopulations was associated with clinicopathological markers as well as Overall Survival (OS) and Disease-Free Survival (DFS). In multivariate analysis, high frequency of CD45RO + cells in IM were confirmed as an independent prognostic marker for DFS (*p* = 0.007, HR = 4.968) and OS (*p* = 0.007, HR = 4.957). Similar findings were observed in the multivariate analysis of the combined frequency of CD45RO+cells in IM and CT.

**Conclusion:**

TILs are associated with patients clinicopathological features. Also, our findings indicate that CD45RO + TILs are a valuable marker for risk prediction in larynx SCC and could predict patients' outcomes.

## Highlights

• TILs may control tumor or delay the acquiring of aggressive phenotypes.
• Higher number of TILs in primary tumor tissues are associated with favorable prognosis.
• Higher number of CD45RO+TILs may be an indicator of efficient anti-tumor immune responses.
• CD45RO+ cells are a valuable biomarker for predicting prognosis of larynx SCC patients.

## Introduction

larynx Squamous Cell Carcinoma (LSCC), the eleventh most common malignancy, is the second most common site of squamous cell carcinoma in head and neck area.^1^ Despite developments in treatment modalities, the 5-year overall survival of LSCC patients showed no improvements for decades and stood at about 60 percent level.^2^ Given the low survival rates, it is necessary to improve the methods of risk prediction to identify patients with higher risk of recurrence and adapt proper treatment approaches for these patients.

The Tumor Immune Microenvironment (TIM) is composed of the immune cells that are infiltrated into the Tumor Microenvironment (TME) during tumor growth. Several reports have shown that TIM has a significant role in cancer progression and outcome.^3^ TIM consists of various immune cell populations like lymphoid and myeloid cells associated with tumor invasion or regression. Tumor-Infiltrating Lymphocytes (TILs) are the most important component of TIM, and in several cancer types, including colorectal cancer, breast cancer, and lymphoma have been reported as a potential prognostic marker with a comparable or greater prognostic significance than conventional TNM staging.^4^ Recent reports indicate the greater prognostic value of TILs when their subpopulation are considered.^5^ The most comprehensive studies in this era were conducted in colorectal cancer and led to introduction of Immunoscore, a new immune-based scoring method.^6^

Head and Neck Squamous Cell Carcinomas (HNSCC), including LSCC, are highly infiltrated by immune cells. The prognostic role of TILs in LSCC has been evaluated in a number of studies.^7^, ^8^, ^9^ However, their results are controversial, which could be due to using different methodological approaches, tumor sites, and TILs subpopulations. Different subsets of lymphocytes show different or even opposite functions in the TME.^10^ T lymphocytes are the principal components of TILs. Cytotoxic T-cells (CTLs) can directly target and destroy tumor cells, and in many cancer types, the presence of CD8+ lymphocytes in the TME has been reported to be linked to improved prognosis.^11^, ^12^ CD4+cells have different subsets and play an important role in directing adaptive immune responses. Studies have shown they can exert both anti-tumor and tumorigenic activities.^13^ CD45RO + memory T-cells, other important component of TIM, have shown positive effects on cancer outcome in several cancer types.^14^

In the present study, we aimed to study the infiltration of CD3+, CD4+, CD8+ and CD45RO+TILs in primary LSCC and assess their correlation with clinicopathological parameters of the disease and patients’ outcomes.

## Methods

### Patients

The study population was selected among patients with larynx SCCs who had surgically removed their primary tumors at Khalili Hospital affiliated to Shiraz University of Medical Sciences, Shiraz, Iran, between July 2013 and March 2017. Patients with the following criteria were included into the study: (i) Patients treated primarily with surgery as their first-line treatment, (ii) Patients with a primary tumor without any evidence of distant metastasis, (iii) Patients with complete clinicopathological data, (iv) Minimum follow-up of at least two years for alive patients. Patients who did not have any of the aforementioned criteria were excluded. Hematoxylin and Eosin (H&E) stained sections of tumors were reviewed by an expert pathologist to confirm histological diagnosis and select Formalin-Fixed and Paraffin-Embedded (FFPE) tissue blocks with both Center of Tumor (CT) and Invasive Margin (IM) regions. Finally, 63 patients with appropriate FFPE tissues and complete pathological and follow up data were included in the study. The experimental protocol of this study was approved by the Ethics Committee of Shiraz University of Medical Sciences (IR.SUMS.REC.1399.840).

The clinical characteristics of patients are shown in Table 1. The mean follow-up period was 47.11 months (range, 18.53–70.06 months). During this time, 14 cases (22.2%) experienced recurrence and 14 cases (22.2%) had cancer-caused death. According to the 7^th^ edition of American Joint Committee on Cancer (AJCC),^15^ majority of patients were of TNM stage III (85.7%,). None of the patients had distant metastases.

**Table 1:** Clinicopathologic characteristics of the patients.

Characteristic	Nº cases (%)
Age, mean (range)	60.19 (39–87)
Sex	
Male	62 (98.4%)
Female	1 (1.6%)
Smoking history	
Smoker	53 (84.1%)
Non-smoker	8 (12.7%)
Missing	2 (3.2%)
T-Stage	
I	0 (0%)
II	5 (7.9%)
III	54 (85.7%)
IV	4 (6.3%)
N Stage	
0	38 (60.3%)
I	8 (12.7%)
II	8 (12.7%)
III	1 (1.6%)
Missing	8 (12.7%)
TNM Stage	
I	(0 %)
II	5 (7.9%)
III	54 (85.7%)
IVA (invaded to the nearby tissues)	4 (6.3%)
Degree of differentiation	
Well-differentiated	16 (25.4%)
Moderately differentiated	31 (49.2%)
Poorly differentiated	15 (23.8%)
Missing	1 (1.6%)
Total	63

### Tissue preparation and immunohistochemistry

Four serial sections, each with 3 μm thickness, were prepared from each FFPE tissue block. Sections were baked for 15 min in 61 °C, then deparaffinized in xylene and rehydrated through decreasing graded ethanol solutions. Antigens were retrieved using high pH antigen retrieval buffer (tris-EDTA, pH9) in a presser cooker for 20 min. Endogenous peroxidase activity was blocked by 3% H_2_O_2_. To block non-specific hydrophobic protein-protein interactions, 10% goat serum was applied. Then tissue sections were incubated with primary antibodies against CD3 (MAD-004072R/D, clone EP41, RTU), CD4 (MAD-004072R/D, clone EP204, RTU), CD8 (MAD-004072R/D, clone SP16, RTU), and CD45RO (Dako, M074201–2, Clone UCHL1, 1/200 dilution) for 45 min. Visualization was performed using Master Polymer Plus HRP detection kit (MAD-00023QK) according to the manufacture instruction. After that, slides were counterstained with hematoxylin to stain nucleus. Finally, the specimens were dehydrated by immersing slides in increasing graded ethanol solutions, washed with xylene and mounted. Normal tonsil samples were used as positive controls for each marker.

### Digital micro-photograph acquisition and positive cell quantification

Micrographs were taken by an expert pathologist who was blinded to patients' characteristics and outcome. For each patient CD3 marker was considered as reference slide to select IM and CT regions with adequate tumor cells and highest immune cell infiltration. Then digital micrographs of other markers (CD4, CD8, and CD45RO) were captured from the same region as of CD3 slides. An optical microscope equipped with Olympus DP2 digital camera was used to acquire images in high-power magnification of 200×. Necrotic regions and areas with crush and other artifacts were excluded. Quantification of TILs staining was performed semi-automatically using ImageJ software. The software was employed to count the number of positive-stained cells per area unit.

### Statistical analysis

All statistical analyses were conducted by SPSS version 21 and Graphs were generated by GraphPad Prism 9.0. All of the clinicopathological features of the study population were categorized as binary variables and differences in TILs infiltration between them were studied by Mann-Whitney *U* test. Cut-off points were determined using Receiver Operating Characteristic (ROC) curves and were calculated based on the survival status. Each subpopulation of stained TILs was dichotomized into low and high based on its corresponding cut-off value. Overall Survival (OS) and Disease-Free Survival (DFS) were defined as the interval time between surgery and cancer-caused death and recurrence, respectively. Patients with no event were censored at the date of their last follow-up. Univariate and Multiple Cox regression analysis was used to investigate the prognostic significance of the studied variables on patient’s OS and DFS. Kaplan-Meier (KM) curves were plotted to illustrate prognostic differences among patient groups; p-values of less than 0.05 were considered significant.

## Results

### CD3+, CD4+, CD8+, and CD45RO + TILs staining patterns

**Figure 1:**
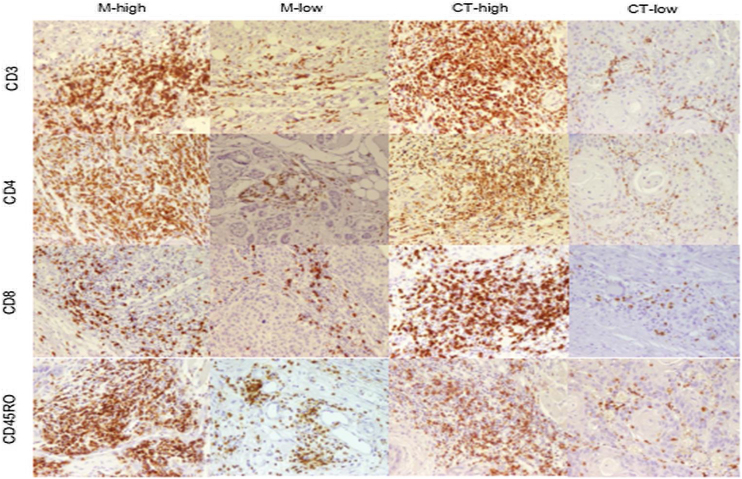
**Immunohistochemical staining of CD3+TILs in HNSCC.** Representative examples of high and low infiltration of CD3, CD4, CD8, and CD45RO positive cells in tumor margin and center of larynx squamous cell carcinoma. Original magnification ×200.

Quantification of the lymphocytes that were infiltrated into tumor tissues showed overall higher infiltration of TILs in IM than CT. However, the differences were not statistically significant. While CD45RO + TILs were the most frequent immune cells in both CT and IM, CD8+ ones constitute the least frequent immune cells (Supplementary Table 1). Fig. 1 illustrate staining pattern of selected markers.

### Association of TILs infiltration with clinicopathological parameters

**Figure 2:**
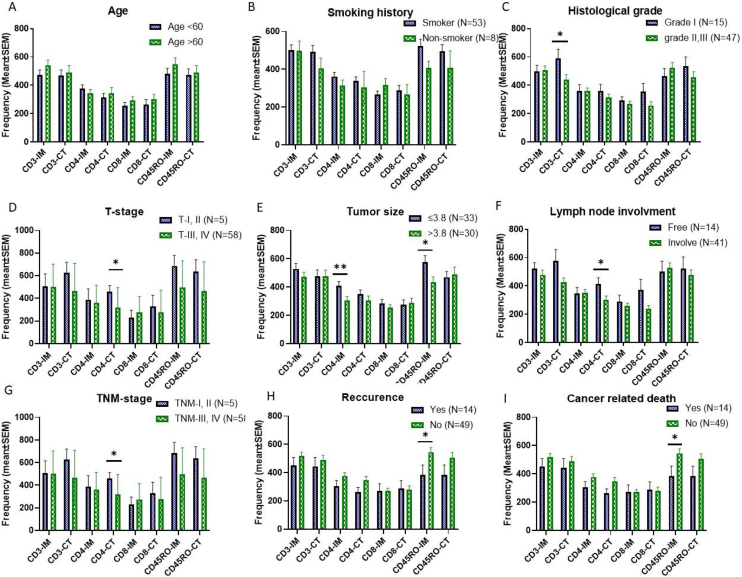
**The mean frequencies of immune cells in tumor tissue of LSCC patients of different clinicopathological features.** There were not significant frequency differences between patients with different age and smoking history (A & B). The frequency of CD3 positive cells in the CT of poorly differentiated tumors was significantly higher than those of well/moderately differentiated tumors (C). CD4 and CD45RO positive cells’ frequency in the IM of tumors with small size were significantly higher than tumors with larger size (E). The frequency of CD4 positive cells in the CT of lymph node involved patients were significantly higher than those of lymph node free ones (F). The frequency of CD4 positive TILs in CT of both TNM-stage I, II and T-I, II tumors were significantly higher than advanced tumors (D & G). The frequency of CD45RO positive cells in IM of patients with history of recurrence and cancer related death was significantly lower than patients without recurrence and death (H & I). The data is presented as mean ± SEM. *Difference is significant at 0.05 level (2-tailed), **Difference is significant at 0.01 level (2-tailed).

According to the results, number of CD3+ TILs in CT of poorly differentiated tumors were significantly higher than those of well/moderately differentiated tumors (*p* = 0.042). Regarding tumor size, 33 (52.4%) patients had smaller tumors and 30 (47.6%) had larger tumors than mean size. Infiltration of CD4+ and CD45RO+ TILs in the IM of small tumors were significantly higher than the opposite group (*p* = 0.009 and *p* = 0.016, respectively). Infiltration of CD4+TILs was higher in CT of patients with lymph node metastasis (*p* = 0.015). Comparison of TILs in different T-stages showed higher infiltration of CD4+ (*p* = 0.043) in CT of primary tumors of T-stage I/II. In samples from TNM-stage I/II patients, number of CD4+ TILs in CT (*p* = 0.043) were significantly more than those of TNM-stage III/IV patients. Detailed information has been presented in Fig. 2.

### Prognostic significance of CD3+, CD4+, CD8+, and CD45RO + TILs on patient’s OS and DFS

First, we analyzed the mean frequency of immune cell infiltration between alive and expired patients as well as patients with and without recurrence by use of Mann-Whitney *U*-test. Among all studied sub-populations, number of CD45RO + cells were considerably higher in IM of patients who had not experienced recurrence (*p* = 0.015) and cancer related death (*p* = 0.015) (Fig. 2 H&I).

For survival analysis, we determined optimal cut-off points of each subpopulation of TILs to stratify the study population into low and highly infiltrated tumors. Next, we performed univariate analysis to evaluate prognostic value of TILs with respect to survival outcomes. Univariate Cox proportional hazards showed that patients with low CD4+TILs in IM of their primary tumors had shorter OS and DFS times with Hazard Ratios (HR) of 4.279 (95% CI 1.193–15.352, *p* = 0.026), and 4.102 (95% CI 1.144–14.714, *p* = 0.030) in comparison to patients with highly infiltrated tumors. Also, CD45RO+ cell in IM (OS: HR = 5.081, 95% CI 1.589–16.241, *p* = 0.006, DFS: HR = 5.099, 95% CI 1.593–16.321, *p* = 0.006) and CT (OS: HR = 3.787, 95% CI 1.185–12.090, *p* = 0.025, DFS: HR = 4.034, 95% CI 1.264–12.877, *p* = 0.019) were significantly associated with OS and DFS (Supplementary Fig. 1 & 2). In order to find any possible prognostic role of TILs, we also performed our analyses using the total number of infiltrated cells, which was calculated by summing up the number of TILs in IM and CT. Among total number of TIL subpopulations (combined frequency in CT and IM), total number of CD4+cells showed a significant association with OS and DFS (HR = 4.130, 95% CI 1.294–13.178, *p* = 0.017, and HR = 3.835, 95% CI 1.202–12.231, *p* = 0.023 respectively). In addition, total CD45RO + TILs had significant trend toward improved OS (low vs. high, HR = 4.054, 95% CI 1.410–14.390, *p* = 0.011) and DFS (low vs. high, HR = 4.788, 95% CI 1.499–15.296, *p* = 0.008). There was no significant association between clinicopathological markers and survival times. Detailed information has been shown in Table 2.

**Table 2:** Univariate Cox regression analysis for disease free survival (DFS) and overall survival (OS) of patients with larynx SCC^1^.

Variable		DFS					OS				
		β	S.E	HR	95% CI for	p-value	β	S.E	HR	95% CI for	p-value
Age	≤60			1(Ref)					1(Ref)		
	>60	-0.058	0.540	0.944	0.327−2.721	0.915	−0.088	0.54	0.916	0.318‒2.641	0.871
Grade	I, II			1(Ref)					1(Ref)		
	III	0.361	0.593	1.434	0.448−4.589	0.543	0.354	0.597	1.425	0.442‒4.593	0.553
Tumor size^a^	≤3.80			1(Ref)					1(Ref)		
	>3.80	0.781	0.558	2.184	0.731‒6.523	0.162	0.853	0.558	2.346	0.786‒7.007	0.127
Lymph node invasion	Positive			1(Ref)					1(Ref)		
	Negative	−0.112	0.592	0.894	0.280‒2.854	0.850	−0.177	0.593	0.838	0.262‒2.677	0.765
Smoking	Yes			1(Ref)					1(Ref)		
	No	0.717	0.652	2.048	0.571‒7.353	0.271	0.647	0.652	1.909	0.532‒6.849	0.321
CD3_IM	High			1(Ref)							
	Low	0.671	0.558	1.957	0.656‒5.843	0.229	0.729	0.558	2.073	0.694‒6.191	0.191
CD4_IM	High			1(Ref)							
	Low	1.412	0.652	4.102	1.144‒14.714	0.030	1.454	0.652	4.279	1.193‒15.352	0.026
CD8_IM	High			1(Ref)							
	Low	0.383	0.558	1.467	0.492‒4.379	0.492	0.451	0.558	1.569	0.525‒4.689	0.420
CD45RO_IM	High			1(Ref)							
	Low	1.629	0.594	5.099	1.593‒16.321	0.006	1.625	0.593	5.081	1.589‒16.241	0.006
CD3_CT	High			1(Ref)							
	Low	0.613	0.558	1.846	0.619‒5.512	0.272	0.638	0.558	1.893	0.634‒5.655	0.253
CD4_CT	High			1(Ref)							
	Low	1.005	0.592	2.731	0.856‒8.712	0.090	0.972	0.592	0.378	0.118‒1.208	0.101
CD8_CT	High			1(Ref)							
	Low	0.171	0.540	1.186	0.412‒3.420	0.752	0.104	0.540	1.109	0.385‒3.199	0.848
CD45RO_CT	High			1(Ref)							
	Low	1.395	0.592	4.034	1.264‒12.877	0.019	1.331	0.592	3.787	1.185‒12.090	0.025
CD3-total	High			1(Ref)							
	Low	0.770	0.558	2.159	0.723‒6.444	0.168	0.806	0.558	2.240	0.750‒6.687	0.149
CD4-total	High			1(Ref)							
	Low	1.344	0.592	3.835	1.202‒12.231	0.023	1.418	0.592	4.130	1.294‒13.178	0.017
CD8-total	High			1(Ref)							
	Low	−0.992	1.040	0.340	0.048‒2.848	0.450	−0.859	1.039	0.423	0.055‒3.246	0.408
CD45RO-total	High			1(Ref)							
	Low	1.566	0.593	4.788	1.499‒15.296	0.008	1.505	0.593	4.504	1.410‒14.390	0.011

Cut-off values: CD3-IM = 513.5; CD4-IM = 353; CD8-IM = 298.5; CD45RO-IM = 441; CD3-CT = 441; CD4-CT = 286.5; CD8-CT = 240; CD45RO-CT = 388.5; CD3-total = 932; CD4-total = 605; CD8-total = 521; CD45RO-total = 833.

SCC, Squamous Cell Carcinoma; S.E, Standard Error; HR, Hazard Ratio; IM, Invasive Margin; CT, Center of Tumor; CI, Confidence Interval.

a Separated by mean.

1 The number of patients with T and TNM stages I,II were low and therefore could not be included in the analyses.

**Figure 3:**
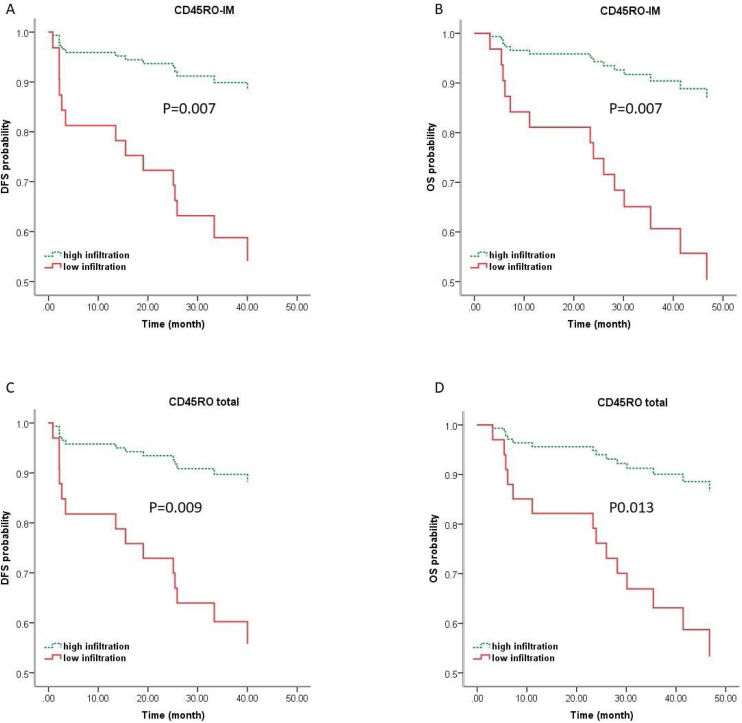
**Kaplan-Meier curves of CD45RO+TILs for OS and DFS prediction of LSCC based on multiple Cox regression survival model.** Kaplan-Meier curves of DFS (A & C) and OS (B & D) of LSCC from multiple analyses indicate that patients with high CD45RO + TILs infiltrates exhibit a significantly improved survival. OS, Overall Survival; DFS, Disease Free Survival; IM, Invasive Margin; CT, Center of Tumor.

For Multivariate analysis, survival models were adjusted for clinically relevant features, including age, tumor grade, tumor size, lymph node status, and TILs subpopulations with p-values less than 0.2. In multiple Cox regression model with backward elimination of non-significant variables at the 5% level, CD45RO + TILs in IM were the only independent prognostic factor that could predict both OS (HR = 4.957, 95% CI 1.150–15.847, *p* = 0.007) and DFS (HR = 4.968, 95% CI 1.552–15.904, *p* = 0.007). With respect to the total number of TILs, total CD45RO + TILs were an independent prognostic marker for OS (HR = 4.390, 95% CI 1.374–14.027, *p* = 0.013) and DFS (HR = 4.663, 95% CI 1.460–14.898, *p* = 0.009) (Fig. 3). Detailed information has been shown in Table 3, Table 4.

**Table 3:** Multiple Cox regression analysis for Disease-Free Survival (DFS) and Overall Survival (OS) of patients with larynx SCC.

Variable		DFS				
		β	S.E	HR	95% CI for HR	p-value
Lymph node invasion	Positive			1(Ref)		
	Negative	−0.104	0.664	0.901	0.245‒3.311	0.875
Age	≤60			1(Ref)		
	>60	0.112	0.583	1.119	0.357‒3.507	0.847
Tumor size^a^	≤3.80			1(Ref)		
	>3.80	0.160	0.642	1.173	0.333‒4.127	0.804
CD4_CT	High			1(Ref)		
	Low	0.614	0.725	1.847	0.446‒7.648	0.397
Grade	I, II			1(Ref)		
	III	−0.731	0.665	0.482	0.131‒1.773	0.272
CD4-IM	High			1(Ref)		
	Low	0.809	0.704	2.245	0.565‒8.916	0.250
CD45RO-CT	High			1(Ref)		
	Low	0.926	0.626	2.526	0.741‒8.612	0.139
CD45RO-IM	High			1(Ref)		
	Low	1.603	0.594	4.968	1.552‒15.904	**0.007**
		**OS**				
Lymph node invasion	Positive			1(Ref)		
	Negative	−0.138	0.701	0.871	0.220‒3.443	0.844
Age	≤60			1(Ref)		
	>60	0.133	0.597	1.142	0.355‒3.677	0.824
Tumor size	≤3.80			1(Ref)		
	>3.80	0.178	0.648	1.195	0.335‒4.259	0.784
CD45RO-CT	High			1(Ref)		
	Low	0.935	0.942	2.547	0.402‒16.155	0.321
CD3-IM	High			1(Ref)		
	Low	−0.509	0.742	0.601	0.140‒2.574	0.493
Grade	I, II			1(Ref)		
	III	0.538	0.647	0.584	0.164‒2.077	0.406
CD4-IM	High			1(Ref)		
	Low	0.787	0.710	2.198	0.547‒8.837	0.267
CD4-CT	High			1(Ref)		
	Low	0.911	0.592	2.486	0.778‒7.938	0.124
CD45RO-IM	High			1(Ref)		
	Low	1.601	0.593	4.957	1.150‒15.847	**0.007**

SCC, Squamous Cell Carcinoma; S.E, Standard Error; HR, Hazard Ratio; CT, Tumor Center; IM, Invasive Margin; CI, Confidence Interval.

a Separated by mean.

**Table 4:** Multiple Cox regression analysis for Disease-Free Survival (DFS) and Overall Survival (OS) of patients with larynx SCC with total variables.

Variable		DFS				
		β	S.E	HR	95% CI for HR	p-value
Age	≤60			1(Ref)		
	>60	0.123	0.560	1.131	0.378‒3.387	0.825
CD3-total	High			1(Ref)		
	Low	−0.241	0.760	0.786	0.177‒3.483	0.751
Tumor size^a^	≤3.80			1(Ref)		
	>3.80	0.263	0.629	1.300	0.379‒4.461	0.676
Lymph node invasion	Positive			1(Ref)		
	Negative	−0.494	0.619	0.610	0.181‒2.051	0.424
Grade	I, II			1(Ref)		
	III	−0.953	0.646	0.386	0.109‒1.368	0.140
CD4-total	High			1(Ref)		
	Low	0.800	0.648	2.226	0.625‒7.935	0.217
CD45RO-total	High			1(Ref)		
	Low	1.540	0.593	4.663	1.460‒14.898	0.009
		**OS**				
Age	≤60			1(Ref)		
	>60	0.098	0.558	1.102	0.369‒3.290	0.861
CD3-total	High			1(Ref)		
	Low	−0.194	0.776	0.824	0.180‒3.770	0.803
Tumor size	≤3.80			1(Ref)		
	>3.80	0.242	0.633	1.273	0.368 ‒4.402	0.702
Lymph node invasion	Positive			1(Ref)		
	Negative	−0.629	0.618	0.533	0.159 ‒1.791	0.309
Grade	I, II			1(Ref)		
	III	−0.869	0.640	0.420	0.120‒1.470	0.175
CD4-total	High			1(Ref)		
	Low	0.896	0.661	2.450	0.671‒8.951	0.175
CD45TO-Total	High			1(Ref)		
	Low	1.479	0.593	4.390	1.374‒14.027	0.013

SCC, Squamous Cell Carcinoma; S.E, Standard Error; HR, Hazard Ratio; CI, Confidence Interval.

a Separated by mean.

## Discussion

Recently, TILs have been introduced as indicators of host immune response against tumor cells and are considered as histopathological features of solid tumors, prognostic biomarkers, and targets for immunotherapy.^16^, ^17^, ^18^ In the present study prognostic value of TILs and their correlation with clinicopathological markers were assessed in larynx SCCs.

Histological grade is one of the clinically important factors in cancer patients. Our results showed a trend of higher infiltration of TILs in poorly differentiated tumors, especially CD3+cells in IM. In HNSCC tumors, Badr et al. found that presence of intra-epithelial TILs levels was positively correlated with high-grade tumors.^19^ Phenotypic differences between poorly differentiated tumor cells and normal cells may lead to increased stimulation of immune cells and consequently their higher recruitment into TME. However, Weenink et al. found that higher infiltration of CD8+TILs in high-grade glioma was associated with higher chemo-attractants, not immunogenic antigens.^20^ High grade tumors have an increased level of angiogenesis.^21^, ^22^ In this regard, increased angiogenesis and higher chemo-attractant secretion by these tumors may explain another reason for higher infiltration of immune cells in poorly differentiated tumors. In our study, other aggressive phenotypes of the disease, including advanced TNM-stage, T-stage and increased tumor size were associated with lower infiltration of TILs subpopulations. Other studies reported that early T-stage was associated with higher infiltration of CD4+TILs.^23^ These findings may indicate that infiltrated T-cells can be indicators of host anti-tumor immune status and higher number of TILs may control tumor or delay the acquiring of aggressive phenotypes. However, it should be mentioned that there is a complexity about association of TILs with clinicopathological markers.^24^ In some cases, clinicopathological markers that are indicators of poor prognosis are associated with higher immune cells infiltration. For instance, in our study patients with lymph node metastases had higher infiltration of CD4+TILs in CT. Because of plasticity in CD4+T-cells, they potentially can act as both anti-tumor and pro-tumor components in TME.^25^, ^26^ Thus this finding may be due to tumor promoting activity of CD4+TILs in TME.

It is proposed that higher number of TILs in primary tumor tissues are associated with favorable prognosis. Previous studies have suggested that TILs in different tumor regions like IM or CT have various functions and distinct prognostic values.^6^ In our study, CD45RO+TILs showed strong prognostic effect on patients’ OS and DFS. In univariate analysis CD4+ and CD45RO+TILs in IM and CD45RO+TILs in CT as well as total number of CD4+ and CD45RO+ cells had prognostic effects on disease outcomes. Prognostic power of CD45RO+TILs was even a bit stronger than that of CD4+TILs. CD4+cells can stimulate cytotoxic activity or, in contrast, suppress the immune response. Because of positive relation between CD4+TILs and tumor outcome, it can be inferred that regulatory subsets may be through downregulation of harmful inflammatory reaction and activated CD4+cells with anti-tumor activity and cytokine secretion could control tumor progression. In the multivariate analysis, CD45RO+TILs had strong prognostic significance and CD4+TILs are in lesser importance to predict patients’ outcome and did not show prognostic importance in multivariate analysis. In some cases, CD4+TILs infiltration may consist of large amount of immune suppressor cells including CD4+Foxp3+ regulatory cells and are associated with poor prognosis. So, because of this complexity in CD4+TILs function in tumor area, it can be conferred that these cells could not be a good predictor of prognosis. Enomoto et al. found no association between clinicopathological markers and the frequency of CD45RO. However, they also found an association between higher level of CD45RO+TILs with better OS and DFS.^27^ Prognostic value of CD45RO+TILs also were reported in various cancer types.^28^, ^29^ The exact mechanism of improved survival by CD45RO+ cells is not fully understood. As marker of memory T-cells, the higher number of CD45RO+TILs in primary tumors may be an indicator of the formation of efficient anti-tumor immune responses against tumor cells; after removal of the primary tumor and consequently decrease in its immune inhibitory pressure on immune cells, they may have the potential of eradicating residual and/or recurrent tumor cells. Some of these memory cells migrate to the secondary lymphoid organs and stay there as central memory T-cells. Also, memory cells are long-lived immune cells that after surgical resection of primary tumor have a great potential to evoke a quick response against previously encountered antigens.^30^ Finally, these cells are relatively safe from being suppressed via T-reg cells.^31^ All of these factors indicate greater anti-tumor potential of CD45RO + memory T-cells. However, this study has limitations including being single-centered and limited in the number of samples. To show the reproducibility of the results of this study, multicenter studies with high number of samples are needed.

## Conclusion

Collectively, it can be inferred that CD45RO+ cells are a valuable biomarker for predicting prognosis of larynx SCC patients and their assessments has the potential to be integrated into clinicopathological prognostic models for patients with LSCC. All of these indicate that new therapeutic approaches by focusing on TILs subpopulations can be designed to improve HNSCC patients' outcomes. However, to incorporate TILs in clinical practice, we suggest repeating the same work with larger sample size.

## Conflicts of interest

The authors declare no conflicts of interest.

## Acknowledgments

The present study was part of a MSc thesis written by Yousef Mohammadi which financially supported by a grant from the Shiraz University of Medical Sciences, Shiraz, Iran [Grant No. 99-01-01-22530] and supported in part by the Shiraz Institute for Cancer Research [Grant No. ICR-100-508].
