# Supplementary material for: CD45RO+TILs: cellular biomarkers for larynx squamous cell carcinoma outcome
Source: Braz J Otorhinolaryngol. 2022 Oct 14;88(Suppl 4):S133–42. doi: 10.1016/j.bjorl.2022.09.007 (PMC9756076; doi:10.1016/j.bjorl.2022.09.007)

BJORL-D-22-00223 – Supplementary material

**Supplementary Table 1** Mean frequency of immune cells in the center and invasive margin of LSCC.

|  | **IM** | | | | **CT** | | | |
| --- | --- | --- | --- | --- | --- | --- | --- | --- |
|  | **CD3** | **CD4** | **CD8** | **CD45RO** | **CD3** | **CD4** | **CD8** | **CD45RO** |
| **Mean** | 502.03 | 361.11 | 271.14 | 509.65 | 477.41 | 328.4 | 280.71 | 479.43 |
| **SEM** | 25.521 | 20.253 | 17.690 | 30.078 | 30.735 | 22.415 | 24.637 | 32.360 |
| **Min‒Max** | 85‒1068 | 79‒684 | 31‒730 | 109‒1087 | 62‒1093 | 78‒874 | 21‒933 | 80‒1030 |

CT, center of tumor; IM, invasive margin; SEM, standard error of mean.

**Supplementary Figure 1 Kaplan-Meier curves of CD4+ and CD45RO+TILs for DFS of LSCC patients based on univariate analyses.** Kaplan-Meier curves of DFS of LSCC from univariate analyses indicate that patients with high CD4+TILs (A & D) as well as CD45RO+TILs (B, C, & E) infiltrates exhibit a significantly improved survival. DFS, Disease Free Survival; IM, Invasive Margin; CT, Center of Tumor.


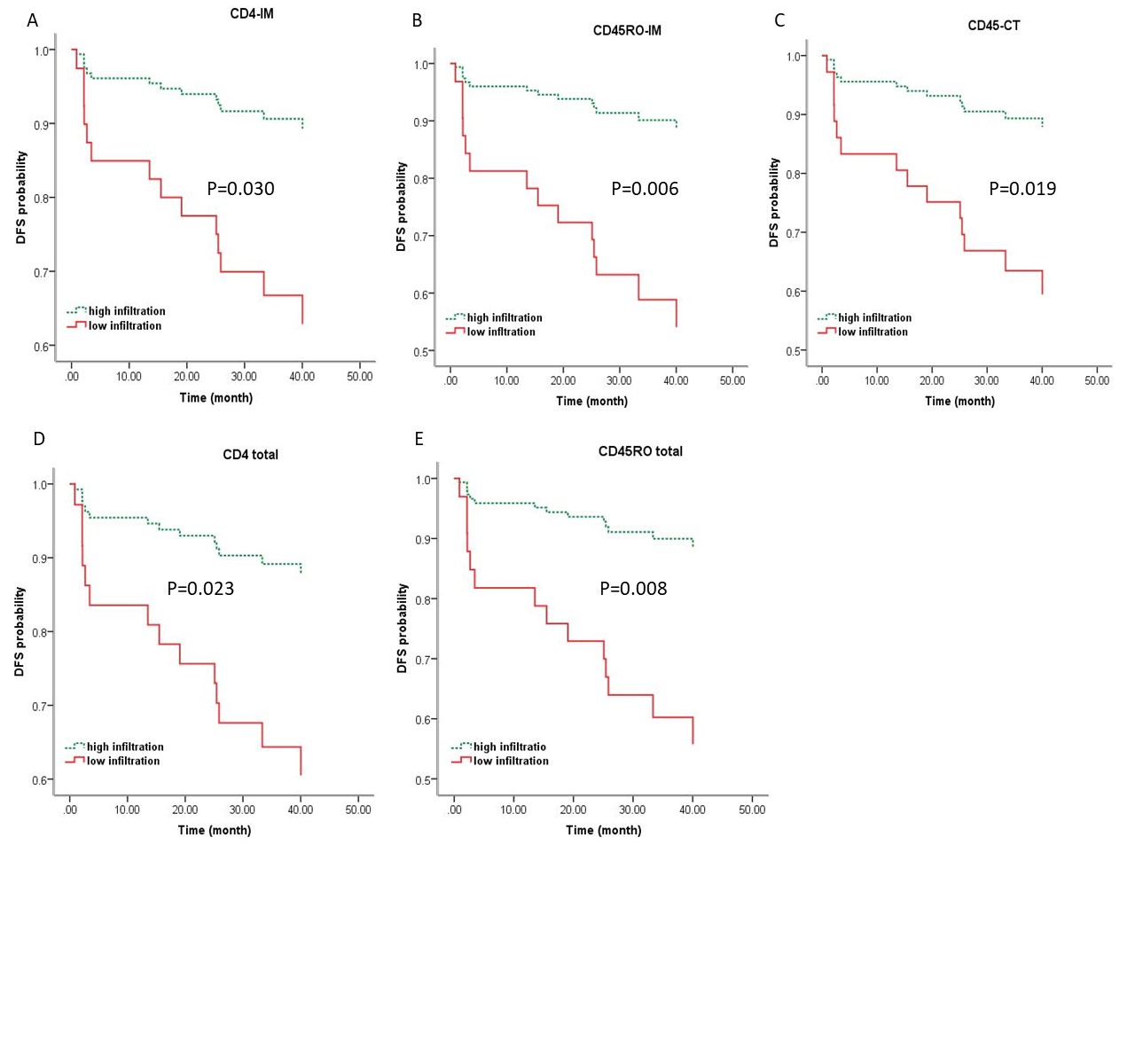


**Supplementary Figure 2** **Kaplan-Meier curves of CD4+ and CD45RO+TILs for OS of LSCC patients based on univariate analyses.** Kaplan-Meier curves of OS of LSCC from univariate analyses indicate that patients with high CD4+TILs (A & D) as well as CD45RO+TILs (B, C, & E) infiltrates exhibit a significantly improved survival. OS, Overall Survival; IM, Invasive Margin; CT, Center of Tumor.


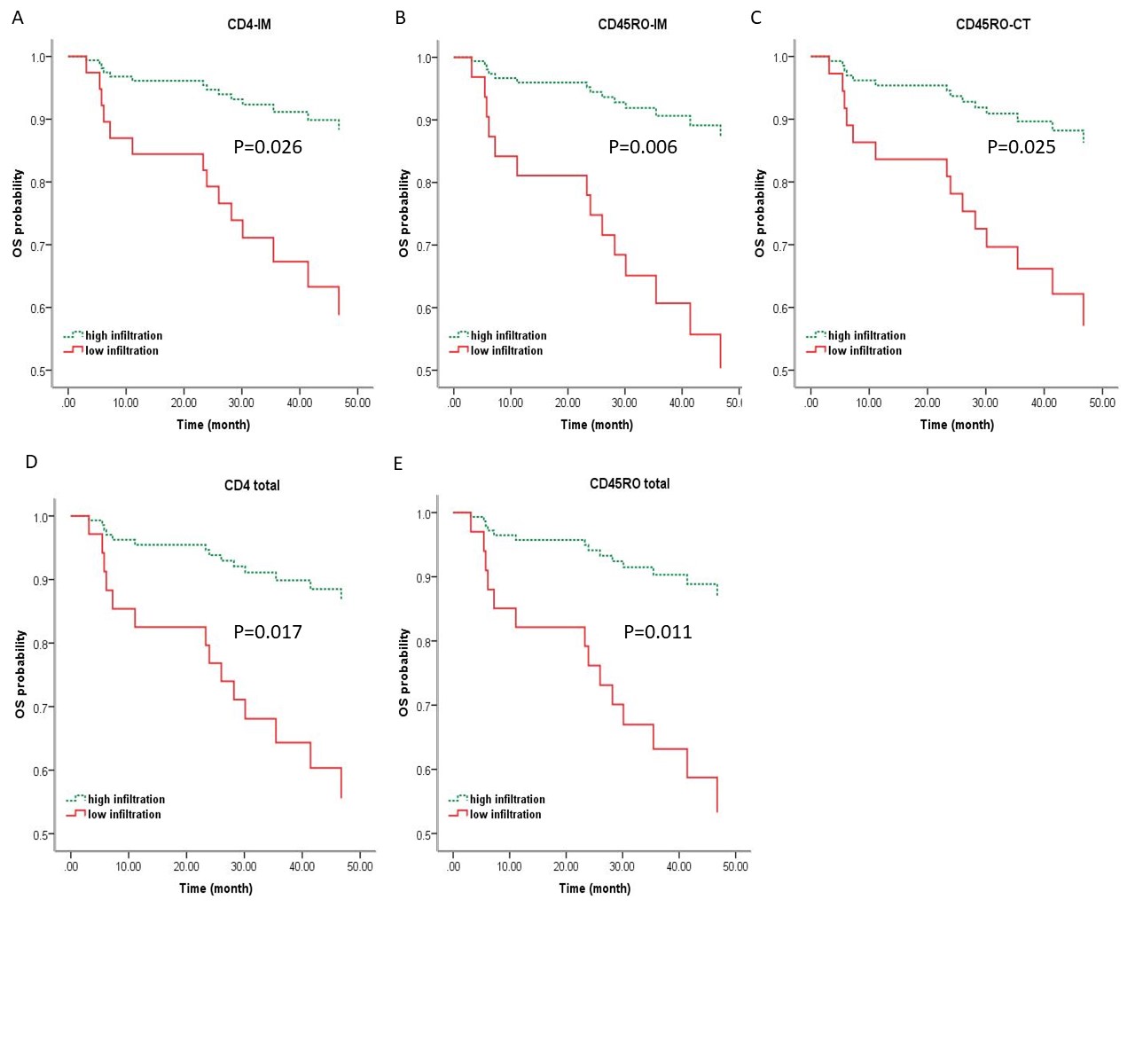

Supplement: Supplementary file 1 [file mmc1.docx]
